# Fabrication of Rapid Electrical Pulse-Based Biosensor Consisting of Truncated DNA Aptamer for Zika Virus Envelope Protein Detection in Clinical Samples

**DOI:** 10.3390/ma16062355

**Published:** 2023-03-15

**Authors:** Moonbong Jang, Myoungro Lee, Hiesang Sohn, Chulhwan Park, Taek Lee

**Affiliations:** 1 Department of Chemical Engineering, Kwangwoon University, Seoul 01897, Republic of Korea; 2 TL Bioindustry, 20 Kwangwoon-Ro, Nowon-Gu, Seoul 01897, Republic of Korea

**Keywords:** truncated aptamer, Zika virus, rapid biosensor, electrical biosensor

## Abstract

Zika virus (ZV) infection causes fatal hemorrhagic fever. Most patients are unaware of their symptoms; therefore, a rapid diagnostic tool is required to detect ZV infection. To solve this problem, we developed a rapid electrical biosensor composed of a truncated DNA aptamer immobilized on an interdigitated gold micro-gap electrode and alternating current electrothermal flow (ACEF) technique. The truncated ZV aptamer (T-ZV apt) was prepared to reduce the manufacturing cost for biosensor fabrication, and it showed binding affinity similar to that of the original ZV aptamer. This pulse-voltammetry-based biosensor was composed of a T-ZV apt immobilized on an interdigitated micro-gap electrode. Atomic force microscopy was used to confirm the biosensor fabrication. In addition, the optimal biosensor performance conditions were investigated using pulse voltammetry. ACEF promoted aptamer-target binding, and the target virus envelope protein was detected in the diluted serum within 10 min. The biosensor waveform increased linearly as the concentration of the Zika envelope in the serum increased, and the detection limit was 90.1 pM. Our results suggest that the fabricated biosensor is a significant milestone for rapid virus detection.

## 1. Introduction

Zika is a flavivirus transmitted through mosquitoes, similar to the dengue virus and yellow fever, and mainly occurs in subtropical climates. After 1950, outbreaks occurred mainly in Africa and some Asian regions [1]. However, cases in French Polynesia (2014) as well as Central America [2] and the Caribbean (2015) have been reported. Owing to the progression of the subtropical climate due to global warming, the habitat of mosquito vectors is expanding, and the distribution and risk of Zika virus is increasing every year [3].

Zika virus infection causes Zika fever with other symptoms, including rash and muscle pain. Mild fever and muscle pain are the primary symptoms. In some pregnant women it causes microcephaly in the fetus [4]. Guillain–Barré syndrome has been reported in the case of an epidemic in French Polynesia [5]. In addition, a recent study suggested the possibility of fatal hemorrhagic fever when infected with a dengue virus of similar structure after Zika virus infection [6]. Conventional diagnostic platforms that detect Zika virus include Zika reverse transcription polymerase chain reaction, Zika antibody test, and Zika plaque reduction neutralization (PRNT). As patients are asymptomatic or show mild symptoms, it is important to conduct a Zika virus diagnostic test when visiting an area where Zika virus is endemic or when suspicious symptoms occur. There is no vaccine or treatment for Zika virus, and a platform that quickly detects Zika virus is required to prevent the possibility of a Zika virus pandemic due to global warming as well as fatal hemorrhagic fever caused by cross-infection with dengue virus. Several biosensors have been developed to detect Zika and dengue viruses. However, it is difficult to use field-ready biosensors because of the complicated detection method and long reaction time [7]. In the field, time is an important factor in detecting Zika virus with high accuracy. Thus, a rapid electrical biosensor based on an aptamer was developed [8,9,10,11]. Zika virus has an envelope protein. These envelope proteins are used as a means of determining the presence or absence of viruses and generally have the large surface area because they surround the substances that make up the virus. Therefore, it is easy to improve signal sensitivity capable of detecting viruses by detecting envelope proteins. Several sensor studies using these envelope proteins have been reported [12,13,14].

Aptamers are short nucleotide sequences that maintain a tertiary structure and have been used as chemical antibodies in biotechnology and various pharmaceutical fields because of their strong binding affinity to specific molecules. Aptamers are attracting attention as an alternative technology for antibodies because they can induce strong binding to the target material and simultaneously solve ethical and economic problems that are associated with antibodies [15]. Aptamers are single nucleic acid strands that are systematically selected through systematic evolution of ligands by exponential enrichment (SELEX) [16,17,18]. An aptamer was used to detect the Zika envelope protein in this study and was selected by target culture, elution of the base sequence, and amplification.

Although studies on aptasensors have already been published several times, there are few cases targeting Zika envelope protein, and in this study a truncated aptamer was used following on from previous studies. In the selected aptamer, the portion that binds to the target molecule is very small [19]. Therefore, to minimize the size of the aptamer, a nucleotide sequence not involved in binding was removed and configured as a truncated aptamer [20]. Minimizing the size of the aptamer not only suppresses unnecessary side effects but also enables continuous amplification through PCR once manufactured. In addition, since it can be produced with less nucleic acid quantitatively, it is possible to supply and purchase aptamers more economically than aptamers used in conventional antibody-based sensors. Therefore, the sensor was configured more selectively and economically by minimizing the aptamer length. Moreover, to apply biosensors in the field (airport, hospital, or other places), the detection time is the main issue in biosensor fabrication. The present study introduces the alternating current electrothermal flow (ACEF) technique for virus detection systems [21]. ACEF induces an electrothermal effect on the electrode, resulting in a local temperature gradient in the microfluid that reacts with the electrode. The non-uniform temperature gradient transforms the shear stress of the fluid and induces micro-flow, which accelerates the response by increasing the probability of coupling events between the electrode surface and the target material. ACEF using microelectrodes promotes the energy dissipation of the fluid through ambient heat conduction, unlike directly heating the fluid to produce a temperature gradient, resulting in a low sample temperature change. Furthermore, as the aptamer and protein buffer solutions used in the biosensor contain salts, they meet the basic ACEF requirements for conductive fluids. Therefore, it reduces the time required for aptamer probe immobilization and protein binding, enabling rapid sensing within 10 min.

**Figure 1:**
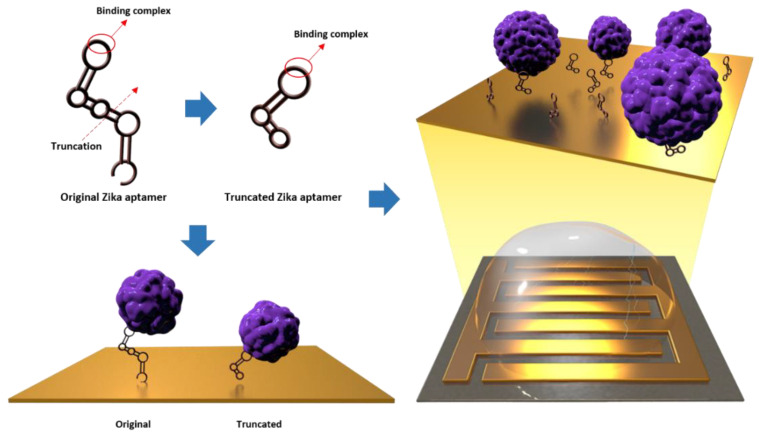
Schematic image of a Zika envelope protein detection biosensor.

Owing to its simplicity and high signal fidelity, an electrical biosensor platform has been introduced to detect Zika virus. Pulse voltammetry measurements were used for electrical detection, and the charge current change was analyzed. Rapid detection of low concentrations of target molecules is a key feature of the pulse voltammetry measurement technique. Cyclic voltammetry is the most commonly used type of voltammetry for electrical detection. It takes approximately 30 min to detect low concentrations of the target molecules [22]. However, using pulse voltammetry and ACEF that increases binding affinity, it is possible to detect a low concentration of the target molecule within 10 min [23]. In addition, to minimize the sample loading volume with repetitive measurements, an interdigitated micro-gap electrode (IDMGE), to which the aptamer probe is fixed, transmits a signal according to the binding of the aptamer to the target protein [24]. IDMGE has a broad area of deviation from the same electrode and improves the sensitivity of electrical biosensors. In previous studies, exosome detection sensors using IDMGE showed a broader linear range and lower detection limit than other sensors. Figure 1 shows a schematic representation of the electrical biosensor. It was manufactured so that the SH group of the truncated aptamer and the gold electrode were combined through a chemical reaction and the Zika envelope protein was specifically combined.

## 2. Experimental Details

### 2.1. Materials

Zika envelope protein was purchased from Sino Biological (Beijing, China), the interdigitated gold electrode used was manufactured by SNI technology (Seoul, Republic of Korea) based on previous research, and the printed circuit board (PCB) combined with the electrode was airlifted from Pulax Korea (Seoul, Republic of Korea). Human serum, horse heart myoglobin, horse heart hemoglobin, and bovine serum albumin were purchased from Sigma-Aldrich (St. Louis, MO, USA). The truncated aptamer and aptamer were synthesized by Bioneer (Daejeon, Republic of Korea).

### 2.2. Truncation of the Zika Virus Envelope Protein Aptamer

The original Zika envelope protein aptamer was developed in our previous study [25]. The aptamer truncation process that followed was as described by Akitomi et al. [26] and was performed by predicting the secondary structure of the sequence and removing unnecessary sequences based on their stem and roof structures. The reported sequence of the Zika envelope aptamer was 5′-TGA CAC CGT ACC TGC TCT AGT GCG CAC TGA ACG ATC CTG CGT CAA GTT CAA GGT TGT GAA GCA CGC CAA GGG ACT AT-3′. It contained primer recognition sequences for 5′-TGA CAC CGT ACC TGC TCT-3′, and 5′-AAG CAC GCC AAG GGA CTA T-3′. Based on this, the mfold server (www.unafold.org, accessed on 20 March 2022) was used for the 5′-AGT GCG CAC TGA ACG ATC CTG CGT CAA GTT CAA GGT TGT G-3′ sequence, excluding the original sequence of the aptamer and the primer, along with the aptamer folding buffer. The secondary structure was predicted based on the salt conditions (140 mM Na+ ions and 5 mM Mg^2+^ ions) and room temperature. Next, the nucleotide sequences forming the loop structure of the original sequence and the primer-excluded sequence were compared, and a common sequence involved in the formation of each loop among the four loops of the original sequence and the three loops of the primer-excluded sequence was identified (GTTGGA, ACGA). Under the condition that the dG value of the predicted primer-excluded sequence did not change, the remaining sequences that did not form a loop at the 5′ and 3′ ends were removed. The performance of the improved Zika aptamer was evaluated using 8% TBE-PAGE and the Kd values were calculated using a bead-based fluorescence binding assay. In 8% TBE-PAGE, Zika and dengue envelope proteins, myoglobin, hemoglobin, and bovine serum albumin (BSA) were used as controls to compare band changes due to the binding of aptamers and proteins. Bead-based fluorescence coupling analysis measures the fluorescence intensity of the bead by adding 6-FAM fluorescence dye to the 5′ end of the aptamer and reacting a magnetic bead with a constant concentration of the Zika envelope protein. The fluorescent aptamer was prepared at four concentrations of 3 nM, 30 nM, 300 nM, and 1 µM, and the beads and fluorescent aptamer were reacted by gently stirring for 1 h, after which the unbound aptamer was removed using deionized water (DIW) and the beads were recovered. Fluorescence intensity of the recovered beads was analyzed at 480 nm absorbance peak and 528 nm divergence peak, which are the fluorescence conditions for FAM dye, using a BioTek Synergy LX multimode reader (CA, USA). Finally, based on the residual fluorescence, Kd of the aptamer was calculated using the adsorption isotherm equation.

### 2.3. Zika Biosensor Fabrication by ACEF-Enhanced

IDMGE was manufactured by sputtering, photolithography, and wet etching to obtain a gap of 5 µm based on a previous study [24]. The width and length of the electrode were 10 μm and 85 μm, respectively, with 5 µm gap between electrodes. For further electrical analysis, IDMGE was attached on PCB chip, and each electrode was connected with sockets using Au wire. The thiol (SH)-modified Zika aptamer and Zika envelope protein were sequentially immobilized on the IDMGE surface. While immobilizing the probes and targets, ACEF was performed for 10 min with 3 V of inputting voltage and 1 Mhz of interval frequency to enhance the self-assembly of immobilization targets [22,27]. After the ACEF steps, residuals were washed and dried using DIW and N2 gas, respectively.

### 2.4. Surface Investigation by Atomic Force Microscopy (AFM)

Characterization of the biomolecules immobilized on the Au electrode surfaces was performed for Au bare electrodes, Au/aptamer electrodes, and Au/aptamer/protein electrodes in non-contact mode using atomic force microscopy (XE7, Park Systems, Seoul, Republic of Korea). PPP-NCHR (Park Systems, Republic of Korea) was used as a cantilever in this analysis, and a silicon tip with a resonant frequency of 330 kHz and spring constant of 42 N/m was selected. Conditions for atomic force microscopy measurement were established based on previous studies [28].

### 2.5. Pulse Voltammetry and Analysis

For pulse voltammetry, the modulation of the charge current of the sensing device was obtained using an electrical measurement system. The system consisted of a power supply (Keysight E3631A DC power supply), a function generator (Tektronix AFG3021C), an operational amplifier (OPAMP), and an oscilloscope (Keysight EDUX1002A oscilloscope). The patterned step pulse from the function generator was applied to the working electrode with a pulse width of 100 µs and pulse amplitude of 0–1 V. The OPAMP was used to convert the output current into output voltage while fixing the counter electrode grounded through the virtual ground. Pulse voltammetry based on an inverting amplifier was performed using the electrical system mentioned previously. Using a DC power supply of 12 V under the 25 V setting condition, −12 V under the −25 V setting condition was supplied to the inverting amplifier, and the function generator that generates the square waveform was set to output 1 V for 100 µs, which is half of the period of 200 µs; the waveform was monitored through an oscilloscope and the LabView program. When the Zika aptamer combines with the target protein on the surface of the IDMGE, the waveform of the function generator changes. The measurements were repeated ten times. In the electric double layer formed in the sensing procedure, the charging current rapidly decreases after the potential step. Therefore, the signal was detected by integrating the voltage value in the range of 0.0004 to 0.0007 s corresponding to the point where the charge current decreased from the point where the potential step started. Abundant negative charges along the aptamer structure, which approach the proximity of the electrode surface, created a charging current from pulse voltammetry events. As the target binds, the aptamer conformation changes, and the bonded target reduces the electrical signal through disturbs the electron condition.

## 3. Results and Discussion

### 3.1. Investigating the Reactivity of the Zika Envelope Aptamer

**Figure 2:**
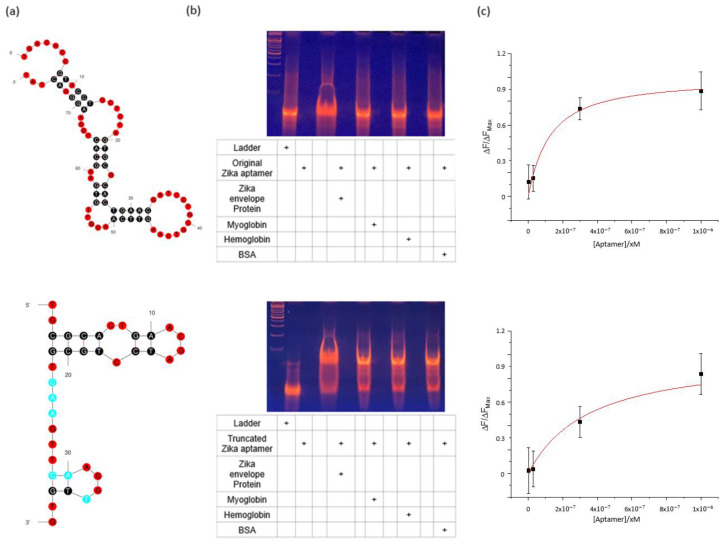
Comparison of the original and truncated Zika aptamers (**a**) Base structures of the original and truncated zika aptamers. (**b**) Result of 8% TBE-PAGE for the original and truncated Zika aptamers. (**c**) Binding affinity of the original and truncated Zika aptamers.

Sequencing of the truncated Zika aptamer was performed by confirming the loop structure of the aptamer and removing the residual sequence not involved in binding; the final sequence was 5′-TGC GCA CTG AAC GAT CCT GCG TCA AGT TCA AGG TTG TG- 3′. The original Zika aptamer and truncated aptamer were compared through their secondary structure, 8% TBE-PAGE analysis, and binding affinity measurement (Figure 2a–c). Figure 2a shows the predicted secondary structure of the original as well as truncated Zika aptamers. The DNA band change was most significant in lane 4 with the truncated Zika aptamer and target Zika envelope protein. Only relatively small changes in the DNA band were found in lanes 6, 8, and 10 with control proteins, myoglobin, hemoglobin, and BSA. This result was the same as the experimental result for the original Zika aptamer, confirming the selective binding ability of the aptamer (Figure 2b). In the bead-based fluorescence binding assay, to determine the Kd of the aptamer, a truncated aptamer with FAM dye was added to the 5′ end of the sequence, and magnetic beads bound to the envelope protein at a fixed concentration of 0.15 mg/mL were used. The aptamer and beads were stirred at room temperature for 1 h, the unreacted aptamer was removed, and the FAM fluorescence of the aptamer bound to the bead was measured. Figure 2c shows the fluorescence intensity as a function of aptamer concentration [29]. According to fluorescence intensity, Kd was calculated based on Equation (1), and the Kd of the aptamer was calculated from the measured fluorescence intensity. The Kd of the truncated Zika aptamer was calculated as 333.1 ± 60.3 nM. The truncated aptamer had a slightly higher Kd value than the original aptamer. This is because the structural stability of the aptamer decreases as the length of the sequence is reduced by truncation. However, in selectivity analysis using 8% TBE-PAGE, the truncated aptamer showed the same selectivity and reactivity as the original.

(1) $$\frac{\Delta F}{\Delta F_{\mathrm{Max}}}=\frac{C_{\mathrm{Zika}\;E}}{K_{d}{+C}_{\mathrm{Zika}\;E}}$$

### 3.2. Surface Investigation of Zika Envelope/Aptamer on the Gold Electrode

**Figure 3:**
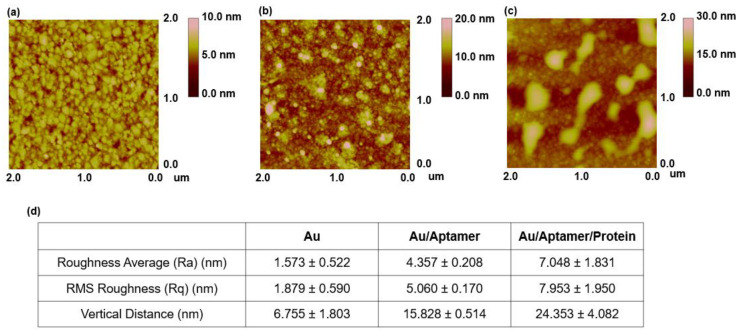
(**a**) AFM image of the bare substrate. (**b**) AFM image of the aptamer on the Au substrate. (**c**) AFM image of the aptamer and Zika envelope conjugate on the Au substrate. (**d**) Surface analysis of the Au substrate, Au/aptamer substrate, and Zika envelope conjugate using roughness average, RMS, and vertical distance.

AFM analysis was performed to determine the average roughness (Ra), root-mean-squared (RMS) roughness (Rq), and vertical distance (Vd) of the sample (Figure 3a–d and Figure S1a–c). The surface of the Au bare substrate is shown in Figure 3a; no other substances were observed on the surface of the substrate. The Ra value of the Au substrate was 1.573 ± 0.522 nm, while the Rq value was 1.879 ± 0.590 nm. Figure 3b shows the surface of the SH-aptamer-modified Au substrate. The Ra of the aptamer-immobilized substrate was 4.357 ± 0.208 nm, Rq was 5.060 ± 0.170 nm, and the Vd value was 15.828 ± 0.514 nm. The Vd value can be interpreted as the size of the aptamer, and the final length of the aptamer selected through SELEX was 77 mer, while the size of 1 mer was about 0.34 nm, indicating a similar result to the reference. Figure 3c shows the AFM measurement of the surface of the aptamer-immobilized Au substrate that reacted successively with the envelope protein. Unlike that shown in Figure 3b, it can be seen in Figure 3c that the protein was immobilized on the surface in the form of a lump. The substrate had an Ra of 7.048 ± 1.831 nm, Rq of 7.953 ± 1.950 nm, and VD of 24.353 ± 4.082 nm, indicating a larger vertical distance compared to the surface on which only the aptamer was fixed.

### 3.3. Investigation of the Electrical Characteristics of the Zika Sensor

When a voltage is applied to an electrode existing in a state of immersion in an electrolyte by using a pulse voltage measurement method, a charging current is formed by an electrical double layer. At this time, the charging current tends to decrease rapidly after the potential step occurs. Therefore, it shows a curved shape and the voltage value decreases in the waveform observation. The electrical signal was measured by integrating the signal from the time when the potential step was applied to the point where the charging current dramatically decreased. At this time, a study was conducted by observing the change in the signal generated by the binding event on the electrode surface. In monitoring the process, different signals were observed depending on the concentration of the aptamer probe connected to the electrode, the binding of the target protein, and the salt condition of the buffer used for transmitting electrical signals. For pulse voltage measurement to detect the optimal signal, the concentration conditions of the truncation aptamer, which is coupled to the IDE electrode and acts as a probe, are discussed. In addition, the detection signals for several types of buffers were analyzed to select buffers that provided the most stable signal (Tables S1–S5).

**Figure 4:**
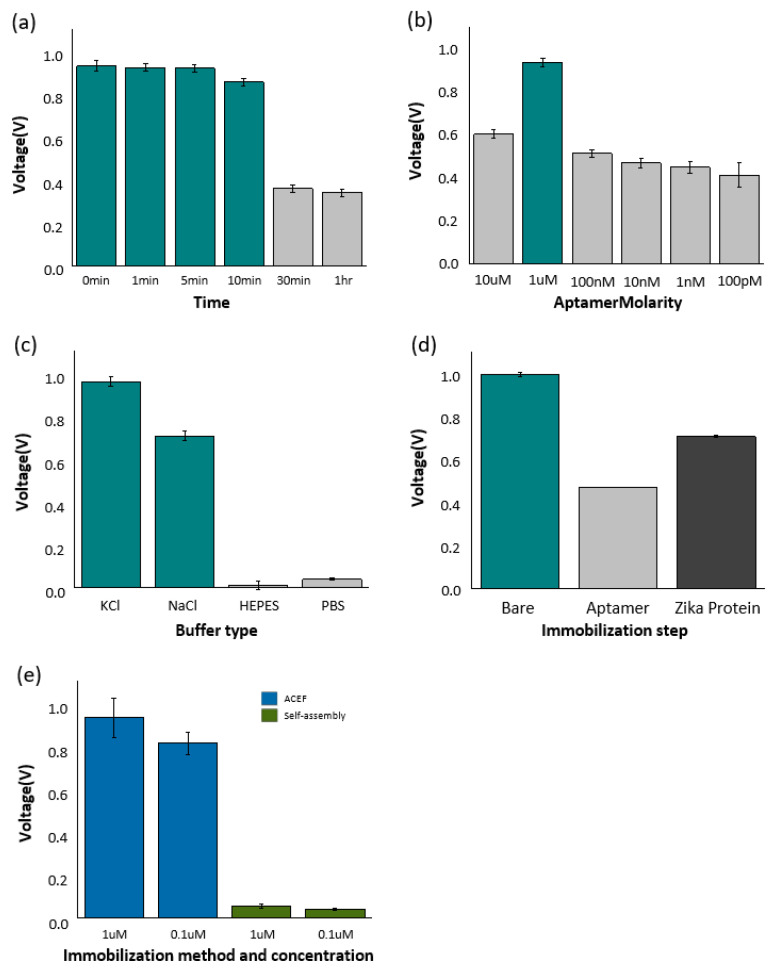
(**a**) Signal changes according to drying time after immobilization of truncated aptamer-Zika envelope protein in electrodes. (**b**) Comparison of electrode signals by truncated aptamer concentration. (**c**) Comparison of signals from electrodes according to buffers used. (**d**) Signal variation by deposition process of truncated aptamer and Zika envelope protein. (**e**) Comparison of ACEF and self-assembly in the immobilization phase of Zika envelope protein at 1 μM and 0.1 μM concentrations.

Figure 4a shows the signal change of the electrode according to the drying time after aptamer-Zika protein immobilization on the electrode. First, the truncated aptamer and zika envelope protein were immobilized before measuring the signal and then dried at intervals of 0 min, 1 min, 5 min, 10 min, 30 min, and 1 h at room temperature. All samples leave at room temperature, and the same concentration and capacity were used for all samples. In the measurement, there was no difference at 0 min, 1 min, and 5 min, and the signal changes from 10 min. After 30 min, it was confirmed that the detection ability of the sensor rather decreased. It is presumed that this was caused by damage to the sample when drying due to the characteristics of the biological sample. Therefore, in this study, the use time of the fixed electrode was set to within 5 min. For ACEF-based aptamer fixation, varying concentrations of the Zika truncated aptamer were prepared by step dilution (Dilute by 10× and in six stages), from 10 µM to 0.1 nM. In measuring the detection ability according to the aptamer concentration, the highest detection ability was confirmed at 1 μM (Figure 4b). Based on these results, 1 μM of Zika truncation aptamer was used for Zika envelope protein detection. For buffer selection, 1 M NaCl, 1 M KCl, 1× PBS, DIW, and HEPES solution, widely known as an electrochemical buffer, were compared. Figure 4c shows the signal changes for each buffer. When using DIW, ions were absent, and no special signal was observed. In addition, only a few signals were detected in the HEPES and 1× PBS solutions. Meanwhile, a high signal was observed in the detection through 1 M NaCl and 1 M KCl solutions, in particular, a more sensitive signal was confirmed in 1 M KCl. Therefore, 1 M KCl (pH 7) was used in the sensing. Figure 4d shows the trend of the sum of voltages observed in the bare, Zika aptamer-coupled, and aptamer-target protein-coupled electrodes. When the aptamer was fixed on the surface of the electrode, the sensor signal tended to decrease. Subsequently, the signal tended to increase slightly as the target protein, Zika envelope protein, combined with the aptamer. An amount of 1 M KCl (pH 7) was used as a buffer solution to measure the signals of the electrodes immobilized with truncated aptamer and Zika envelope protein. When calculated using an amino acid sequence, the isoelectric point (PI) of Zika envelope protein was 6.5. When the truncated aptamer was bound to the bare electrode, the signal was reduced because the resistance increased due to immobilization of the sample on the electrode surface. Furthermore, the increased signal change in combination with the target Zika envelope protein is due to the electrical double layer formed by the combination of truncated aptamer and Zika envelope protein. Figure 4e shows the change in the electrical characteristics of the electrode in the presence or absence of ACEF.

ACEF and self-assembly for immobilizing the diluted Zika envelope protein on the electrode were compared at concentrations of 1 and 0.1 µM. There were no significant differences in voltage in self-assembly. On the other hand, the signal increased significantly on ACEF. Therefore, the results of this study were derived using ACEF.

### 3.4. Sensor Performance Evaluation and Zika Envelope Protein Serum Test

**Figure 5:**
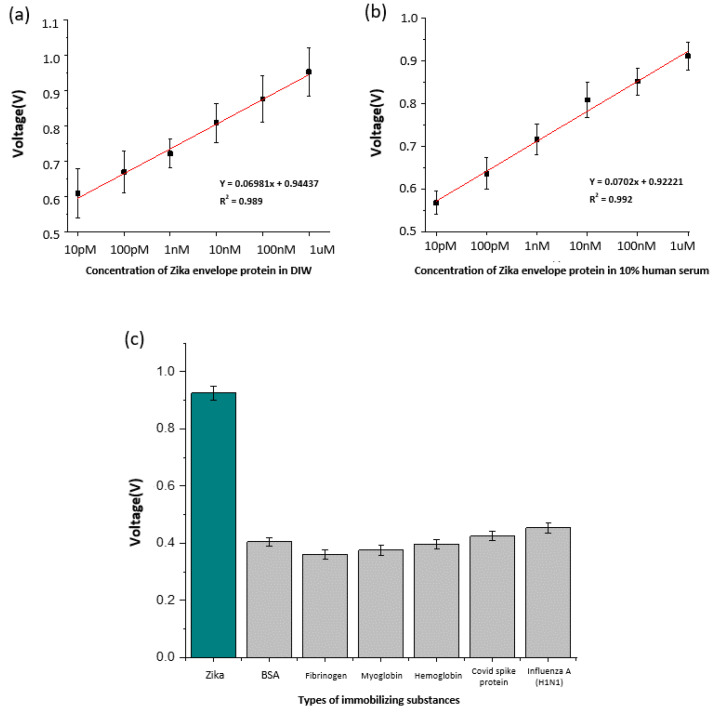
(**a**) Calibration curve according to the Zika envelope protein concentration. (**b**) Calibration curve according to the Zika envelope protein concentration in 10% human serum. (**c**) Comparison of the selectivity of Zika envelope protein with bio-derived materials and other virus proteins in the designed electrodes.

To evaluate the sensor performance, the concentration of Zika envelope protein was determined using six stages of dilution, from 1 µM to 10 pM (Tables S6 and S7). As shown in Figure 5a, the signal tended to decrease as the concentration of the sample decreased. An electrical double layer was formed on the electrode surface depending on the concentration of the Zika envelope protein; the higher the concentration, the stronger the double layer formed. To consider the differences in the condition of each electrode, the signal measurement value of the Zika envelope protein was calibrated by dividing it by the BARE value. The detection limit of the sensor was calculated by creating a trend line, based on the signal for each concentration. As shown in Figure 5a, the slope of the sensor was 0.06981, and the limit of detection (LOD) of the sensor calculated based on the graph with an intercept of 0.94437 was 38.49 pM (At S/N = 3).

The LOD was calculated using the Zika envelope protein sample diluted with 10% human serum. As performed previously, measurements were carried out in six steps, 10 times each, in the concentration range of 10 pM–1 µM, and the results are shown in Figure 5b. For 10% human serum, the sensor slope was 0.0702, the intercept was 0.92221, and the sensor LOD was 90.1 pM.

Table 1 shows previous studies targeting Zika envelope proteins and non-structural proteins of the Zika virus. Although there are many methods for detecting Zika virus, the method performed in this study has an economic advantage in that aptamers can be synthesized at a low cost by performing PCR compared to studies using antibodies. In addition, no work requires specialized personnel such as RT-PCR, ELISA, and FICT and, we show results lower than the LOD of other studies using aptamers while targeting the Zika envelope protein. In Table 1, there are studies with excellent results such as LOD and detection range, but this study has a significant advantage in that it can directly detect Zika virus contained in human serum during the sensing process without going through any special preprocessing process. In addition, since ACEF processing enables faster detection than conventional sensors, the sensor designed in this study can be a fast and inexpensive diagnostic platform. Finally, to confirm the selectivity of the sensor, major interfering substances in the blood, such as hemoglobin, myoglobin, fibrinogen, and BSA, were used and covid spike protein and Influenza A (H1N1) were used to identify signals for other viruses (Table S8). The experimental results are shown in Figure 5c. Our sensor showed a high signal in response to the Zika envelope but showed no significant reactivity with other proteins.

## 4. Conclusions

In this study, an aptamer that specifically binds to the Zika virus envelope protein was synthesized and used for the construction of an electric biosensor. In addition, the synthesized aptamer introduced a truncation technique to shorten the length of the base sequence. Accordingly, it is possible not only to save the cost of manufacturing the base sequence that does not affect binding, but also to reduce the time required in the synthesis process. ACEF forms a local temperature gradient on the surface of an electrode, which causes a local flow within the buffer on the electrode. Therefore, the formation of bonds between the electrode surface and the truncated aptamer was significantly increased. Increasing the probability of binding events through the convection of fluids reduces the detection time to less than 10 min, enabling rapid detection. AFM data demonstrate that the synthesized truncated aptamer and the target (Zika envelope protein) bind well. Pulse voltage was measured based on sensors manufactured using ACEF technology. Following this measurement, the Zika envelope protein was detected in 10% human serum within 10 min. In addition, a selectivity test confirmed that the manufactured electrode selectively binds to the target material. As a result, the Zika envelope protein showed a significantly higher signal. Signals excluding the target material were those of the electrode itself, indicating that the manufactured electrode specifically binds to the target material. Therefore, the biosensor proposed in this paper is an excellent model of a virus detection platform that can perform fast detection at a low cost.

## Figures and Tables

**Table 1:** Biosensor comparison table for zika virus detection.

Probe	Detection Method	Target Material	Detection Range	LOD	Ref
Antibody	Electric Chemical	Envelope Protein	10 pM~1 nM	10 pM	[30]
Antibody	Electric	NS1	0.1~100 ng/mL	0.1 ng/mL	[31]
Primer	RT-PCR	NS1	10^3^~10^6^ pfu/mL	337 pfu/mL	[32]
Aptamer	Electric Chemical	Envelope Protein	100 pM~10 μM	93.14 pM	[25]
Aptamer	ELISA	NS1	~10 ng/mL	1 ng/mL	[33]
Peptide Aptamer	FICT	Envelope Protein	0.15~10.92 ng/mL	0.15 ng/mL	[12]
Aptamer	Electric	Envelope Protein	10 pM~1 μM	90.1 pM	This study

## Data Availability

Data sharing not applicable to this article.

## Supplementary Materials

The following supporting information can be downloaded at: https://www.mdpi.com/article/10.3390/ma16062355/s1, Figure S1: Surface analysis of AFM according to immobilization steps, surface analysis of Au substrate (a), surface analysis of Au substrate with Zika truncated aptamer immobilized (b), and surface analysis of Au substrate with Zika truncated aptamer-Zika envelope protein immobilized (c); Table S1: Signal change according to drying time after immobilization of truncated aptamer-Zika envelope protein; Table S2: The measurements result after 10× cascade dilution to select the optimal truncated aptamer concentration; Table S3: The measurements result from various solutions to select the optimal buffer; Table S4: The measurement result of signal change according to the immobilized process of samples in the electrode; Table S5: The measurement result of ACEF and self-assembly in the immobilization phase of Zika envelope protein at 1uM and 0.1uM concentrations; Table S6: The measurement result of concentration-specific signal measurements of Zika envelope protein diluted in DIW on designed electrodes; Table S7: The measurement result of concentration-specific signal measurements of Zika envelope protein diluted in 10% human serum on designed electrodes; Table S8: The measurement result of the selectivity of Zika envelope protein with bio-derived materials and other virus proteins in the designed electrodes.
