# Supplementary material for: Fabrication of Rapid Electrical Pulse-Based Biosensor Consisting of Truncated DNA Aptamer for Zika Virus Envelope Protein Detection in Clinical Samples
Source: Materials (Basel). 2023 Mar 15;16(6):2355. doi: 10.3390/ma16062355 (PMC10054023; doi:10.3390/ma16062355)
Supplement: Supplementary file 1 [file materials-16-02355-s001.zip › materials-2200225-supplementary.pdf]

Supporting Information

Fabrication of rapid electrical pulse-based biosensor consisted of truncated DNA aptamer for zika virus detection in clinical sample

Moonbong Jang1,†, Myoungro Lee1,†, Hiesang Sohn1, Chulhwan Park1, Taek Lee1,2,*

a)

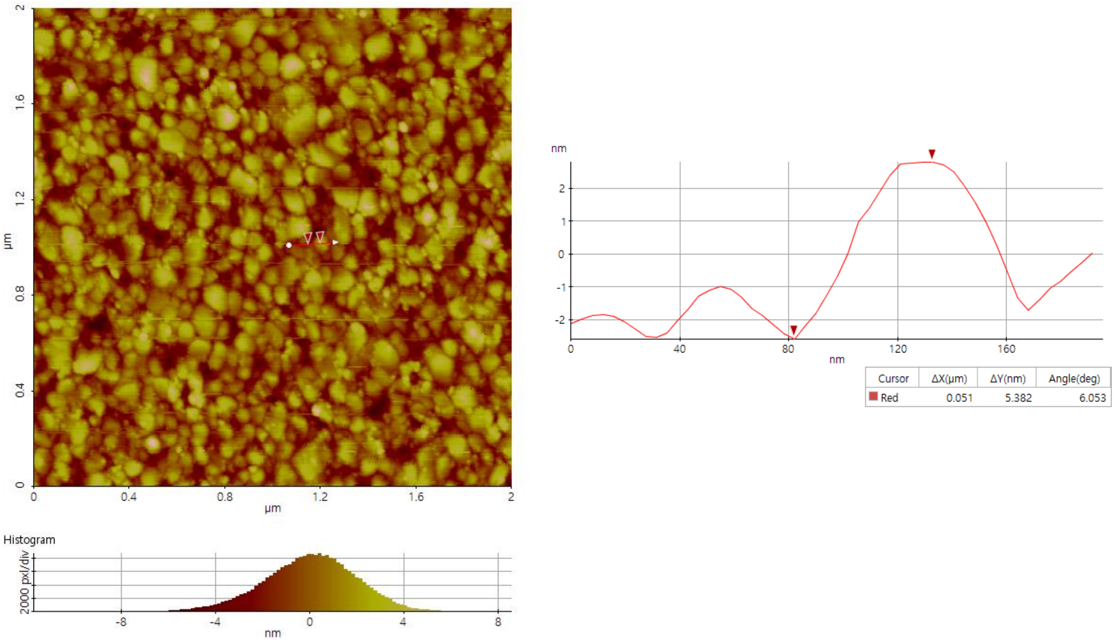

Line	Min(nm)	Max(nm)	Mid(nm)	Mean(nm)	Rpv(nm)	Rq(nm)	Ra(nm)	Rz(nm)	Rsk	Rku
Red	-2.588	2.798	0.105	-0.560	5.387	1.746	1.476	N/A	0.787	2.210

b)

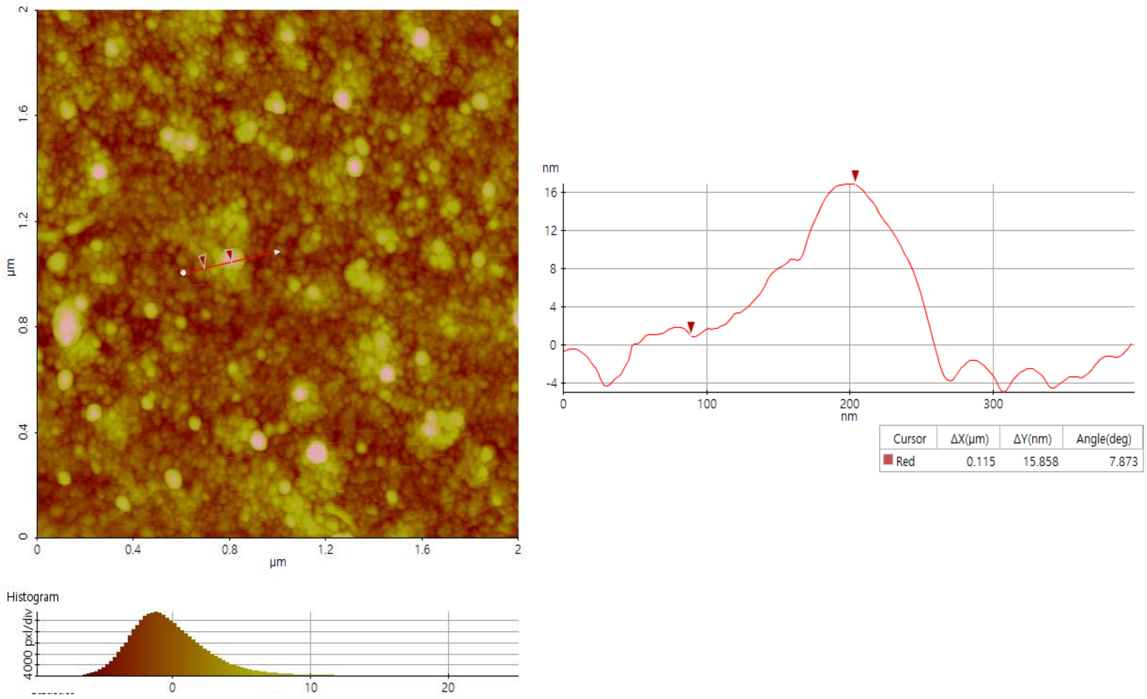

Line	Min(nm)	Max(nm)	Mid(nm)	Mean(nm)	Rpv(nm)	Rq(nm)	Ra(nm)	Rz(nm)	Rsk	Rku
Red	-5.006	16.824	5.909	2.563	21.830	6.500	5.401	N/A	0.918	2.565

c)

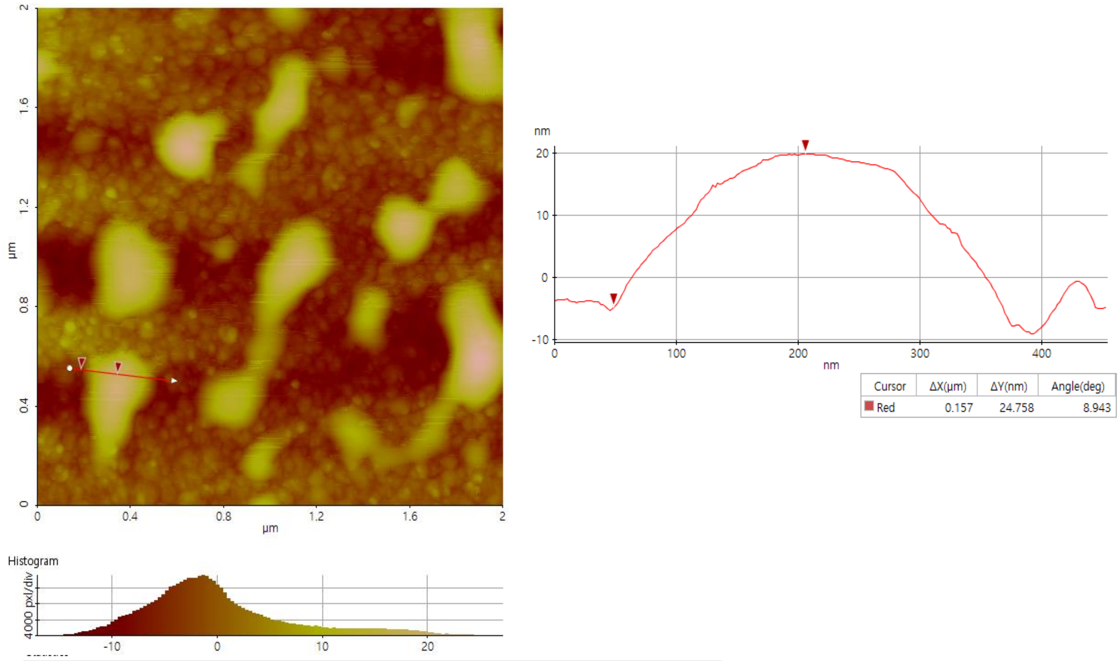

Line	Min(nm)	Max(nm)	Mid(nm)	Mean(nm)	Rpv(nm)	Rq(nm)	Ra(nm)	Rz(nm)	Rsk	Rku
Red	-9.254	19.839	5.293	6.904	29.092	9.924	9.029	N/A	-0.076	1.444

Figure. S1. (a) Vertical analysis of Au substrate, (b) Vertical analysis of aptamer on the Au substrate, (c) Vertical analysis of aptamer and Zika envelope conjugate on the Au.

Table. S1. Signal change according to drying time after immobilization of truncated aptamer-Zika envelope protein.

Time	0 min	1 min	5 min	10 min	10 min	30 min
**Voltage**	0.92558 ± 0.02463	0.91969 ± 0.01541	0.91540 ± 0.01609	0.85085 ± 0.01779	0.35643 ± 0.01735	0.33775 ± 0.01779

Table. S2. Measured values after 10x cascade dilution to select the optimal truncated aptamer concentration.

Molarity	10 uM	1 uM	100 nM	10 nM	1 nM	100 pM
**Voltage**	0.60201 ± 0.01849	0.93367 ± 0.02096	0.51256 ± 0.01911	0.46901 ± 0.02328	0.44891 ± 0.02772	0.41206 ± 0.05707

Table. S3. The measurements result from various solutions to select the optimal buffer.

Buffer	KCl	NaCl	HEPES	PBS
**Voltage**	0.91942 ± 0.02463	0.73695 ± 0.01541	0.08174 ± 0.00928	0.03089 ± 0.00506

Table. S4. The measurement result of signal change according to the immobilized process of samples in the electrode.

Measurement conditions	Bare	Aptamer	Zika Protein
**Voltage**	0.99302 ± 0.07119	0.49832 ± 0.00101	0.70537 ± 0.03351

Table. S5. The measurement result of ACEF and self-assembly in the immobilization phase of Zika envelope protein at 1uM and 0.1uM concentrations.

immobilization condition	1uM ACEF	0.1uM ACEF	1uM	0.1uM
**Voltage**	0.91942 ± 0.02463	0.73695 ± 0.01541	0.08174 ± 0.00928	0.03089 ± 0.00506

Table. S6. Results of concentration-specific signal measurements of Zika envelope protein diluted in DIW on designed electrodes.

Concentration of Zika envelope protein in DIW	10 pM	100 pM	1 nM	10 nM	100 nM	1 uM
Voltage	0.95273 ± 0.06910	0.87616 ± 0.06604	0.80818 ± 0.05508	0.72197 ± 0.04186	0.66939 ± 0.05986	0.60977 ± 0.06981

Table. S7. Results of concentration-specific signal measurements of Zika envelope protein diluted in 10% human serum on designed electrodes.

Concentration of Zika envelope protein in 10% human serum	10 pM	100 pM	1 nM	10 nM	100 nM	1 uM
Voltage	0.91108 ± 0.03324	0.85186 ± 0.03144	0.80819 ± 0.04066	0.71641 ± 0.03632	0.63582 ± 0.03713	0.56750 ± 0.02657

Table. S8. The measurement result of the selectivity of Zika envelope protein with bio-derived materials and other virus proteins in the designed electrodes.

Types of immobilizing substances	Zika envelope protein	BSA	Fibrinogen	Myoglobin	Hemoglobin	Covid spike protein	Influenza A (H1N1)
Voltage	0.92558 ± 0.02464	0.40531 ± 0.01541	0.36075 ± 0.01736	0.37656 ± 0.01780	0.39811 ± 0.01609	0.42585 ± 0.01614	0.45430 ± 0.01856
